# Simultaneous Voltammetric Determination of Epinine and Venlafaxine Using Disposable Screen-Printed Graphite Electrode Modified by Bimetallic Ni-Co-Metal–Organic-Framework Nanosheets

**DOI:** 10.3390/molecules28052128

**Published:** 2023-02-24

**Authors:** Zahra Dourandish, Hadi Beitollahi, Iran Sheikhshoaie

**Affiliations:** 1 Department of Chemistry, Faculty of Science, Shahid Bahonar University of Kerman, Kerman 76175-133, Iran; 2 Environment Department, Institute of Science and High Technology and Environmental Sciences, Graduate University of Advanced Technology, Kerman 7631818356, Iran

**Keywords:** NiCo metal–organic framework, two-dimensional, epinine, venlafaxine, voltammetric sensor

## Abstract

We constructed two-dimensional NiCo-metal–organic-framework (NiCo-MOF) nanosheets based on a facile protocol and then characterized them using multiple approaches (X-ray diffraction (XRD), energy-dispersive X-ray spectroscopy (EDS), field emission-scanning electron microscopy (FE-SEM), and N_2_ adsorption/desorption isotherms techniques). As a sensitive electroactive material, the as-fabricated bimetallic NiCo-MOF nanosheets were employed to modify a screen-printed graphite electrode surface (NiCo-MOF/SPGE) for epinine electro-oxidation. According to the findings, there was a great improvement in the current responses of the epinine because of the appreciable electron transfer reaction and catalytic performance of the as-produced NiCo-MOF nanosheets. Differential pulse voltammetry (DPV), cyclic voltammetry (CV) and chronoamperometry were utilized to analyze the electrochemical activity of the epinine on the NiCo-MOF/SPGE. A linear calibration plot was obtained in the broad concentration range (0.07–335.0 µM) with a high sensitivity (0.1173 µA/µM) and a commendable correlation coefficient (0.9997). The limit of detection (S/N = 3) was estimated at 0.02 µM for the epinine. According to findings from DPV, the electrochemical sensor of the NiCo-MOF/SPGE could co-detect epinine and venlafaxine. The repeatability, reproducibility and stability of the NiCo-metal–organic-framework-nanosheets-modified electrode were investigated, and the relative standard deviations obtained indicated that the NiCo-MOF/SPGE had superior repeatability, reproducibility and stability. The as-constructed sensor was successfully applicable in sensing the study analytes in real specimens.

## 1. Introduction

*N*-methyldopamine, deoxyepinephrine or epinine has catecholamine architecture and serves as a natural organic drug. It is present in animals, plants and insects, a with pharmacological profile identical to that of epinephrine. This medicine is a product of the enzymatic hydrolysis of ibopamine, which acts as a cardiovascular drug in the treatment of congestive heart failure [1,2,3,4,5]. Nevertheless, Barger and Dale reported an increase in blood pressure following the administration of epinine, suggesting its role in increasing the blood pressure [6]. Despite the many advantages of the epinine, the excess use of this drug has detrimental impacts of human health, and, therefore, it should be used under supervision. Accordingly, there is a need for a valid and rapid analytical in vivo approach to detecting epinine in real specimens.

1-(2-(dimethylamino)-1-(4-methoxy-phenyl) ethyl) cyclohexanol, or venlafaxine, belongs to the selective serotonin-norepinephrine reuptake inhibitors, as an antidepressant drug. It can elevate the doses of norepinephrine and serotonin neurotransmitters in the whole body and brain. The mechanism of this drug is to block transporter reuptake proteins for neurotransmitters implicated in mood, which leaves more active neurotransmitters in the synapse [7,8]. Venlafaxine has effects on both noradrenaline and dopamine reuptake. The antidepressant performance of the drug has been attributed to a combination of impacts on the reuptake mechanisms. This drug has a broad spectrum as an antidepressant for adults and children who suffer from nightmares, narcolepsy and cataplexy. The effective daily dose of venlafaxine varies between 75 and 375 mg. Its overdose may be associated with cardiac arrhythmia, depression, serotonin syndrome, hypotension, hypertension, coma and even mortality [9,10,11]. Urine and serum drug concentrations should be monitored clinically to assess for toxicity, complications, interactions and therapeutic effectiveness.

The clinical management of some patients simultaneously with diverse therapeutic drugs emphasizes the necessity of the simultaneous detection of these drugs. Heart rate and blood pressure can increase under the influence of both venlafaxine and epinine, highlighting the need to control the concentration of these drugs in patients’ body fluids.

Various analytical techniques have been used so far to determine the presence of venlafaxine or epinine in different samples, some of which are high-performance liquid chromatography [12,13], spectrophotometry [14], capillary electrophoresis [15,16] and chemiluminescence [17].

Meanwhile, much attention has been directed towards electrochemical sensors in the analysis of drugs, food additives and other biological and environmental specimens in real matrices [18,19,20,21,22]. The compactness and portability of electrochemical equipment make it possible to analyze samples in situ and rapidly without handling the sample pretreatment [23,24,25,26,27,28,29,30].

The electrode material directly affects the electrochemical measurement of the target analytes, which makes clear the importance of the electrode selection for molecular measurement. Screen-printed electrodes (SPE), as planar instruments, consist of three electrodes (working, pseudo-reference and auxiliary) usually applicable in electrochemical cells. These electrodes are effective candidates for producing accurate, versatile and cost-effective sensors in a miniature size. The sample requirement at the microliter level and the repeatability of the response of these electrodes provide an ideal analytical platform for point-of-care sensing [31,32,33]. The surface modification of SPEs with diverse modifiers enhances the electronic transfer, which makes them powerful electroanalytical tools with high sensitivity, a low limit of detection (LOD) and reduced overpotentials [34,35,36]. Electrochemical sensors can be greatly enhanced by modifying the electrode surface with suitable electroactive substances [37,38,39,40,41,42].

A metal–organic framework (MOF) contains organic molecules (linkers or ligands) and metal ions or clusters affected by a coordination bond, and MOFs are an interesting porous and functional substance with tenability in terms of their composition and structure. Some of the main applications of MOFs include gas adsorption, catalysis, separation and substance detection substance, owing to their commendable properties such as their big surface area, manifold topological structures, ease of operation, tenability in terms of porosity and appreciable stability [43,44,45,46,47]. Nanomaterials differ in their electrochemical and catalytic behaviors based on their surface morphologies, shapes and sizes. Various heterometallic MOFs have been fabricated through the integration of one or more metallic nodes into the monometallic framework for the modification and improvement of the properties of monometallic MOFs, such as the enhancement of their electrocatalytic and catalytic performances. Various metallic centers integrated into identical frameworks have shown new defects in the MOF structure and an excellent synergism between diverse metals, thereby enhancing their intrinsic properties, such as their optical, magnetic and electronic profiles [48,49,50,51,52].

The electrocatalytic activity of MOFs with thin sheets is better when compared with that of their bulk counterparts. Binding metal ions with organic linkers form very thin 2D MOF nanosheets. Metal atoms are placed on the surface. The electronic architecture of the metal centers in the MOF is influenced by the organic ligands via a potent interplay. Such admirable properties and a controllable electronic architecture constitute a high potential for electrochemical catalysis and analysis [53,54].

The current attempt was made to develop a facile and easy protocol to fabricate bimetallic MOF nanosheets (NiCo-MOF) as a strong electrode material for electrochemical epinine and venlafaxine sensors. We characterized the as-fabricated bimetallic NiCo-MOF nanosheets using field-emission scanning electron microscopy (FE-SEM), X-ray diffraction (XRD), N_2_ adsorption/desorption isotherms and energy-dispersive X-ray spectroscopy (EDS).

Chronoamperometry, DPV and CV techniques were implemented to analyze the epinine electro-oxidation on the modified NiCo-MOF/SPGE. A commendable electrocatalytic activity was observed for the bimetallic NiCo-MOF nanosheets during the epinine determination because of a remarkable elevation in the voltametric signal of the study analytes. The applicability of the as-developed sensor was explored by using it to sense venlafaxine and epinine in real specimens.

## 2. Results and Discussion

### 2.1. Determinations of NiCo-MOF Nanosheet Profiles

**Figure 1:**
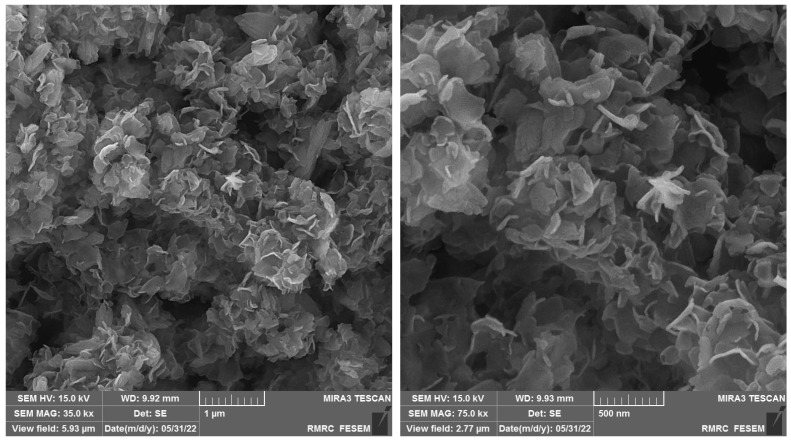
FE-SEM images prepared from as-constructed bimetallic NiCo-MOF nanosheets with different magnifications.

The FE-SEM images (with different magnifications) were captured to explore the as-fabricated NiCo-MOF materials for morphology (Figure 1). The observations verified a hierarchical, multilayered, nanosheets-like structure. A sheet-like nanostructure was found for the NiCo-MOF with a sheet thickness of about 20 nm. The ultra-thin structure is verified by the propensity to create wrinkles on the layers of the nanosheets. A large number of wrinkled sheets allows for further the flexibility of the NiCo-MOF. These nanosheets are intermingled with each other and have formed enough open space between adjacent nanosheets.

**Figure 2:**
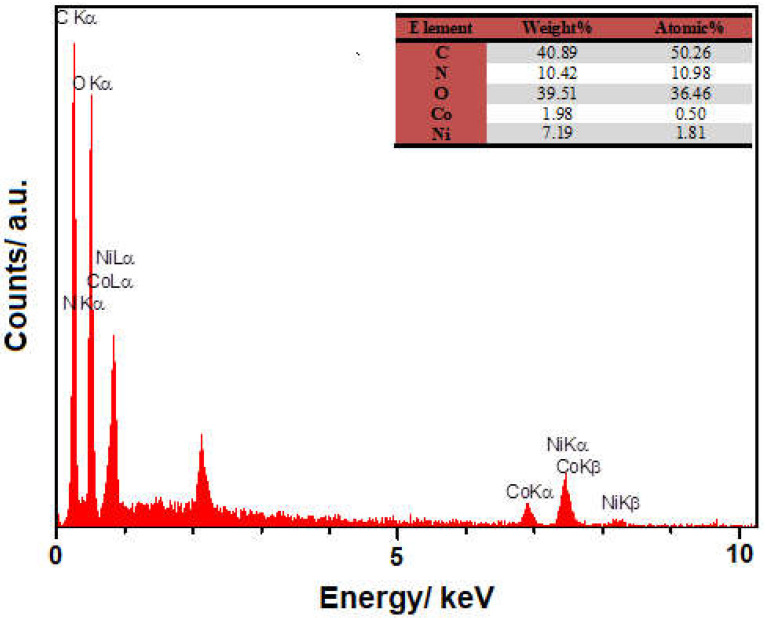
EDS spectrum prepared from as-constructed bimetallic NiCo-MOF nanosheets.

Figure 2 depicts the EDS spectra recorded for elemental analysis of the bimetallic NiCo-MOF nanosheets, the findings of which indicate the presence of C, O, Ni, Co and N elements.

**Figure 3:**
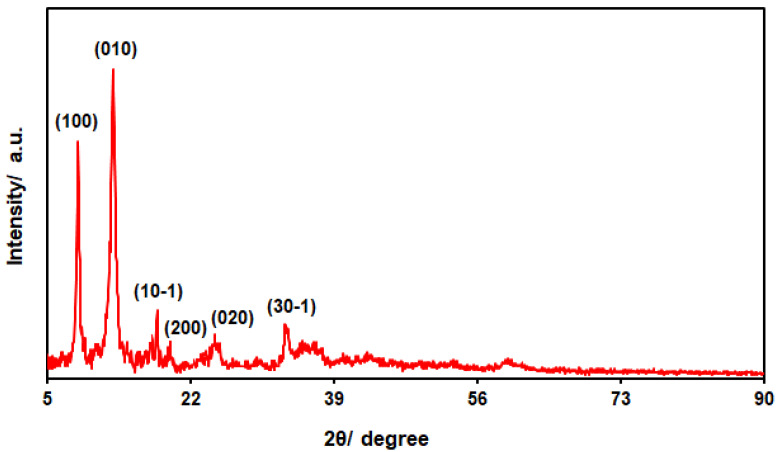
XRD pattern captured for as-fabricated bimetallic NiCo-MOF nanosheets.

Figure 3 depicts the powder XRD patterns for the structural analysis of the bimetallic NiCo-MOF nanosheets, the findings of which indicate peaks at the 2θ angles of 33.2°, 23.8°, 19.4°, 17.6°, 12.7° and 9.3°, attributed to the crystal planes of (30-1), (020), (200), (10-1), (010) and (100), sequentially, in accordance with the standard diffraction pattern of a Ni-MOF [55,56,57,58]. Meanwhile, no other peaks were recorded, highlighting the high phase purity of the bimetallic NiCo-MOF nanosheets.

**Figure 4:**
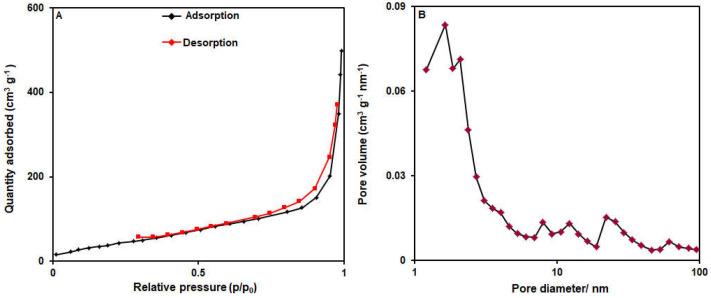
(**A**) N_2_ adsorption/desorption isotherms and (**B**) pore size distribution curves of bimetallic NiCo-MOF nanosheets.

Figure 4 shows the data from the N_2_ adsorption/desorption isotherms for the specification of the surface area and pore size distribution of the bimetallic NiCo-MOF nanosheets. Figure 4A depicts the N_2_ adsorption/desorption curve representing a type IV isotherm along with an obvious hysteresis loop (type H3). Based on the BET data, the surface area of the bimetallic NiCo-MOF nanosheets was estimated at 172.39 m^2^/g, and, based on the BJH data, the mean pore diameter was calculated to be 1.64 nm (Figure 4B).

### 2.2. Electrochemical Activity of Epinine on the NiCo-MOF/SPGE

**Scheme 1:**
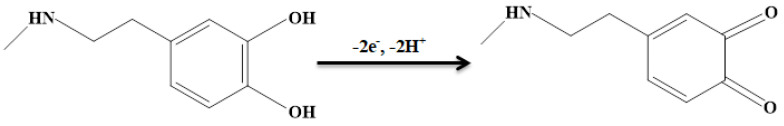
Electro−oxidation mechanism of epinine at NiCo-MOF/SPGE.

**Figure 5:**
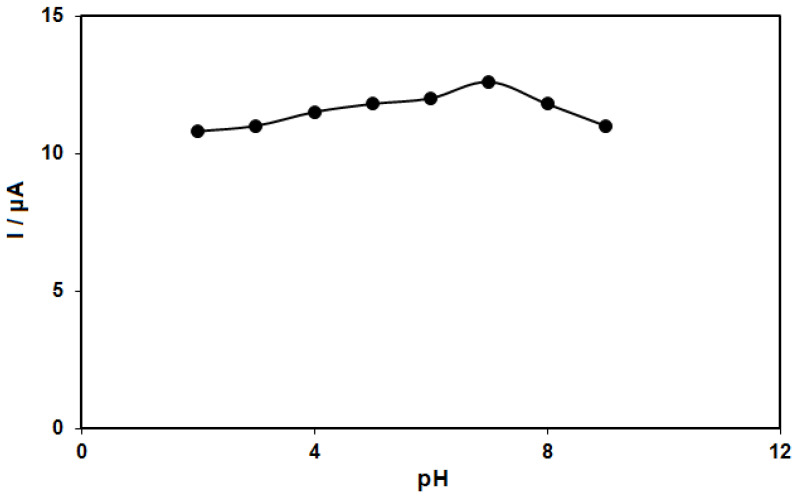
Plot of Ip vs. pH obtained from DPVs of NiCo-MOF/SPGE in a solution containing 100.0 μM of epinine in 0.1 M PBS with different pHs (2.0, 3.0, 4.0, 5.0, 6.0, 7.0, 8.0 and 9.0).

The epinine electro-oxidation has an association with electron and proton exchange (Scheme 1). Thus, the effect of pH on the electrochemical response of the epinine on the NiCo-MOF/SPGE sensor should be evaluated. For this purpose, experiments were performed in 0.1 M PBS at the pH range of 2.0-9.0 using DPV. The results showed a greater epinine oxidation current on the surface of the NiCo-MOF/SPGE in a neutral status relative to in acidic or alkaline conditions, so pH 7.0 was considered as the optimal value for the electro-oxidation of epinine on the as-produced electrode surface (Figure 5).

**Figure 6:**
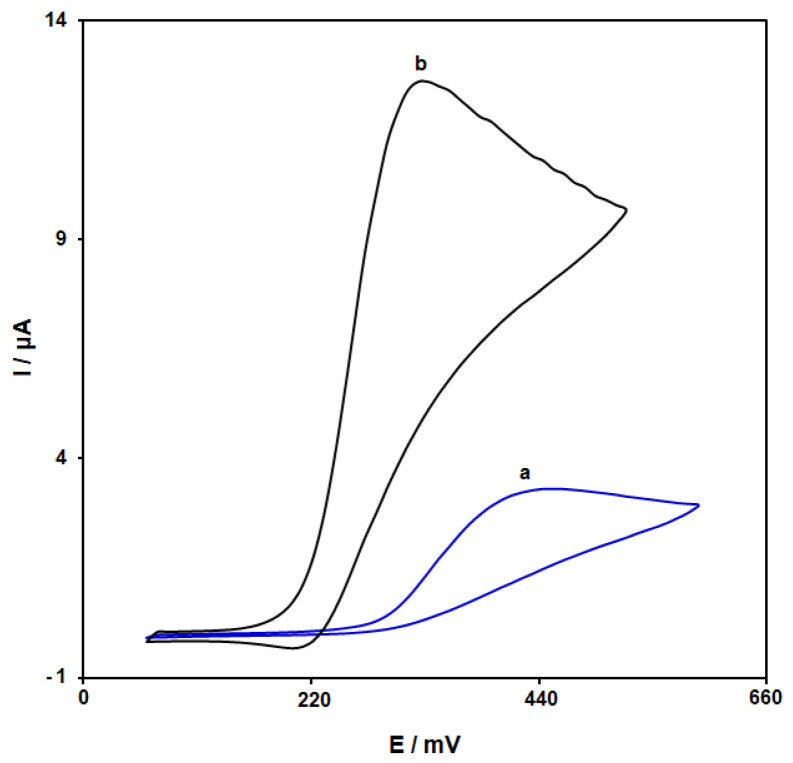
Cyclic voltammograms captured of (a) bare SPGE and (b) NiCo-MOF/SPGE for epinine (100.0 μM) in PBS (pH 7.0; 0.1 M) at scan rate of 50 mV/s.

The cyclic voltammograms (CVs) were captured for the epinine solution (100.0 μM, pH = 7.0) on the bare SPGE (Figure 6, curve a) and the NiCo-MOF-nanosheets-modified SPGE (Figure 5, curve b). The epinine oxidation current on the SPGE surface was estimated to be 3.3 μA. There was an increase in the epinine oxidation current on the NiCo-MOF/SPGE, with a maximum value of about 12.6 μA. Hence, the NiCo-MOF/SPGE had a greater conductivity than the unmodified SPGE, thereby introducing NiCo-MOF nanosheets as highly conductive mediators for SPGE modification in the sensing of epinine.

### 2.3. Analysis of Scan Rate Effect

**Figure 7:**
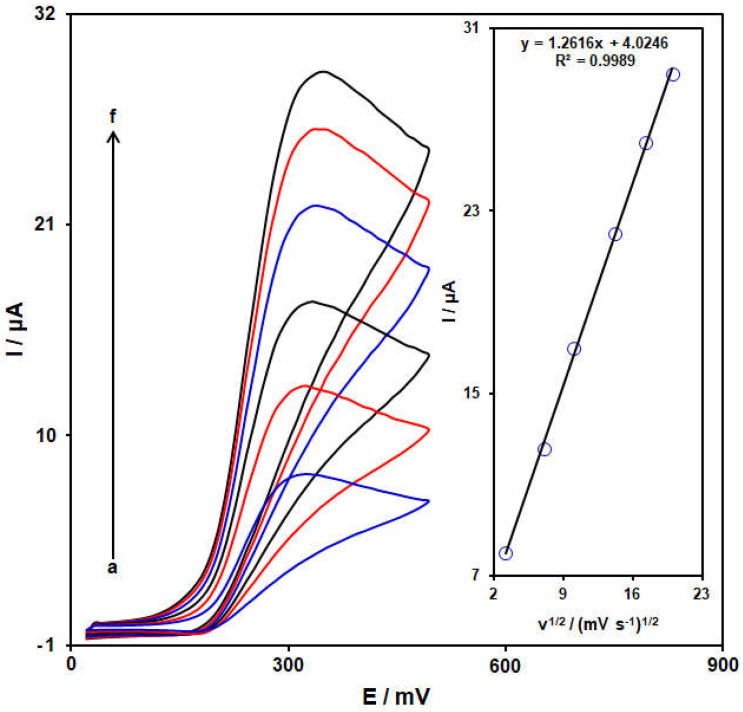
Cyclic voltammograms captured for epinine (100.0 μM) in PBS (pH 7.0, 0.1 M) on NiCo-MOF/SPGE at variable scan rates; a−f correspond to (a) 10, (b) 50, (c) 100, (d) 200, (e) 300 and (f) 400 mV/s. Inset: Plot of Ipa against υ^1/2^ for epinine electro−oxidation.

The electrochemical response of epinine on the NiCo-MOF/SPGE was evaluated using the CV method. Figure 7 shows the CVs captured for epinine (100.0 μM) on the NiCo-MOF/SPGE in PBS (pH = 7.0, 0.1 M) at variables scan rates. Figure 6 (inset) depicts the anodic peak current (Ipa) of the epinine, representing a linear relationship with the scan rate square root (υ^1/2^), Ipa = 1.2616 υ^1/2^ + 4.0246 (R^2^ = 0.9989). Hence, the electrochemical behavior of the epinine follows a diffusion-controlled process.

### 2.4. Chronoamperometric Determinations

**Figure 8:**
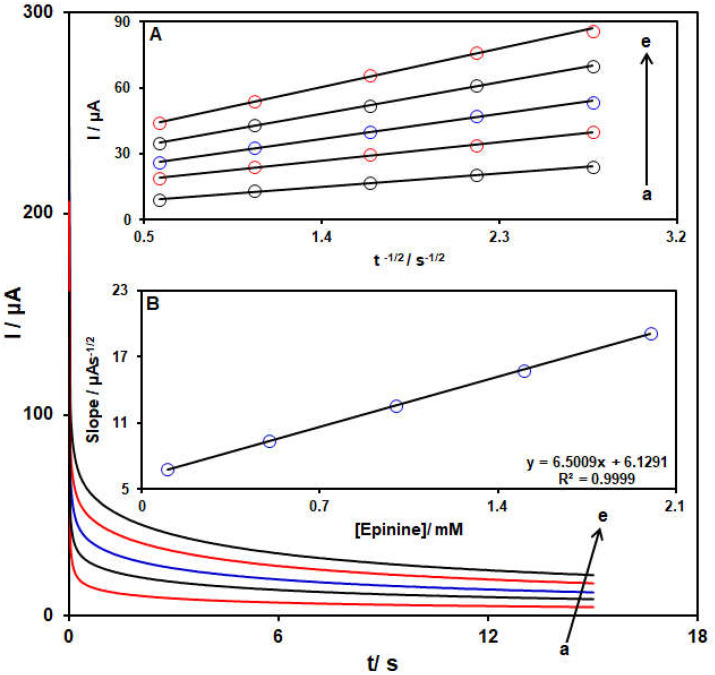
Chronoamperograms captured for the NiCo-MOF/SPGE in the exposure to variable epinine concentrations, a–e correspond to (a) 0.1, (b) 0.5, (c) 1.0, (d) 1.5, and (e) 2 mM of epinine in PBS (pH 7.0, 0.1 M). Insets: (**A**): Plots of I vs. t^−1/2^ from chronoamperograms a–e. (**B**) Plot of straight lines slope vs. epinine level.

Chronoamperometry was used to explore the epinine oxidation on the NiCo-MOF/SPGE surface (Figure 8). Chronoamperometric determinations for variable epinine levels on the modified electrode were carried out at the working electrode potential of 0.38 V. The diffusion coefficient (D) for epinine was measured in aqueous solution in accordance with the Cottrell equation:

I = nFAC_b_D^1/2^ π^−1/2^ t ^−1/2^

Herein, C_b_ stands for the concentration (mol/cm^3^), D for the diffusion coefficient (cm^2^/s), A for the electrode area (cm^2^), F for Faraday’s constant, t for the time (s) and n for the number of transferred electrons. Figure 8A shows the plots of I against t ^−1/2^ for variable epinine contents. Figure 8B indicates the slopes from the straight lines plotted against the epinine levels. The D value was computed to be 3.5 × 10^−6^ cm^2^/s according to the slope of the obtained plots and also the Cottrell equation.

### 2.5. Standard Plot and Limit of Detection

**Figure 9:**
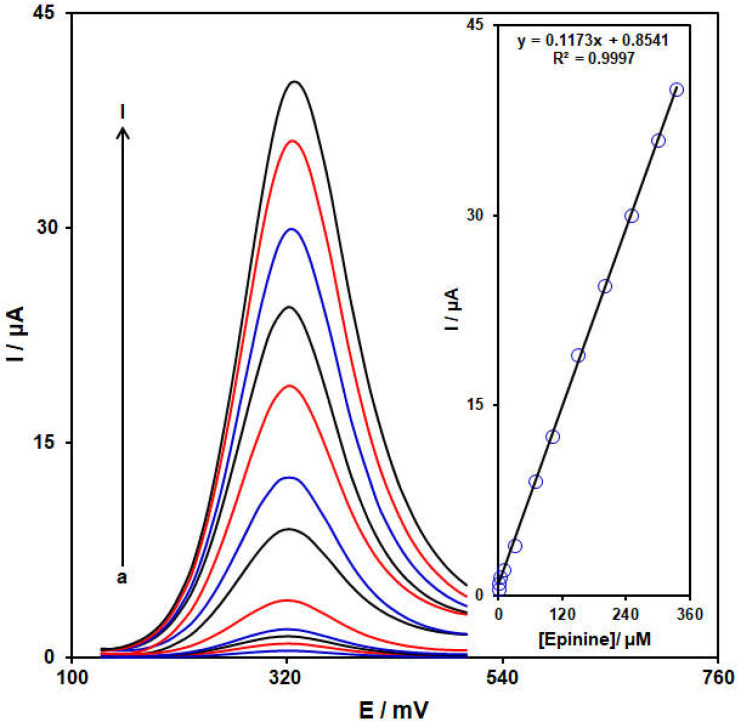
Differential pulse voltammograms captured for the NiCo-MOF/SPGE in the exposure to variable epinine concentrations, a–l correspond to (a) 0.07, (b) 0.5, (c) 2.5, (d) 10.0, (e) 30.0, (f) 70.0, (g) 100.0, (h) 150.0, (i) 200.0, (j) 250.0, (k) 300.0, and (l) 335.0 μM epinine in PBS (pH 7.0, 0.1 M). Insets: Relationships between the Ipa and levels of epinine.

The standard plot was prepared using the DPV approach. Figure 8 illustrates the DPV captured for the NiCo-MOF/SPGE in its exposure to variable epinine levels. An elevation in the concentration of epinine obviously resulted in an increase in the Ipa of the epinine. The standard curve for the variable epinine levels revealed a linear dynamic range as broad as 0.07 to 335.0 μM, with the equation of Ipa = 0.1173C_epinine_ + 0.8541 (R^2^ = 0.9997) (Figure 9, Inset). The sensitivity was calculated to be 0.1173 μM /μA for the NiCo-MOF/SPGE in sensing epinine.

Also, the limit of detection, C_m_, of the epinine was calculated using the following equation:

C_m_ = 3S_b_/m

where m is the slope of the calibration plot (0.1173 μA/μM) and S_b_ is the standard deviation of the blank response, which is obtained from 15 replicate measurements of the blank solution. The limit of detection of the prepared sensor (NiCo-MOF/SPGE) is about 0.02 μM.

### 2.6. Co-Detection of Epinine and Venlafaxine

**Figure 10:**
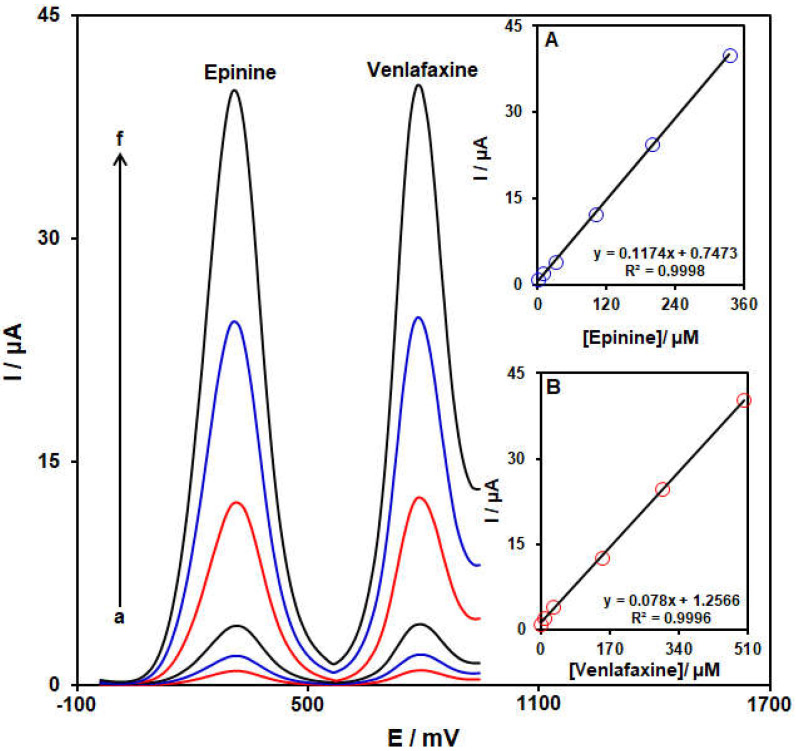
Differential pulse voltammograms captured for NiCo-MOF/SPGE in PBS (pH 7.0, 0.1 M) with variable epinine and venlafaxine levels (a—f): (a) 0.5 + 1.0, (b) 10.0 + 10.0, (c) 30.0 + 30.0, (d) 100.0 + 150.0, (e) 200.0 + 300.0, and (f) 335.0 + 500.0 μM of epinine and venlafaxine level, sequentially. Insets: (**A**): Plot of peak current as a function of epinine levels; (**B**): Plot of peak current as a function of venlafaxine levels.

The current work aimed to fabricate a modified electrode capable of distinguishing the electrochemical behaviors of venlafaxine and epinine. The analytical tests were performed by varying the content of venlafaxine and epinine at NiCo-MOF/SPGE as the working electrode in PBS (pH 7.0, 0.1 M). Differential pulse voltammograms were captured for the NiCo-MOF/SPGE at variable levels of epinine and venlafaxine (Figure 10). Separate oxidation signals appeared at the potentials of about 325 and 795 mV on the NiCo-MOF/SPGE over epinine and venlafaxine, sequentially. The sensitivity of modified electrode relative to epinine in the absence (0.1173 µA/µM, Figure 9) and presence (0.1174 µA/µM, Figure 10A) of venlafaxine was identical, suggesting the independent oxidation of venlafaxine and epinine on the NiCo-MOF/SPGE, and also the feasibility of concurrent determinations of the two analytes with no interference.

### 2.7. Interference Study

The effect of several interference species on the determination of 30.0 µM epinine was studied using DPV. The results showed that the interfering effects of Mg^2+^, K^+^, Na^+^, Ca^2+^ and Cl^−^ ions and several organic species such as tryptophan, vitamin B_6_, vitamin B_9_, phenylalanine and citric acid on the anodic peak current of epinine is less than 5%. Hence, the ability of NiCo-MOF/SPGE to selectively determine epinine in the presence of some compounds is confirmed.

### 2.8. The Repeatability, Reproducibility and Stability Analysis

For practical applications, the repeatability, reproducibility and stability of the electrochemical sensors are essential. The repeatability of the response of the modified electrode (NiCo-MOF/SPGE) was estimated by performing the electrochemical experiment repeatedly (six measurements) with the same NiCo-MOF/SPGE in a buffer solution (0.1 M, PBS) containing 50.0 μM of epinine. The relative standard deviation (R.S.D) based on six replicates was found to be 4.2%, which indicated that the NiCo-MOF/SPGE has good repeatability.

The reproducibility of the method was also evaluated by preparing six modified electrodes (NiCo-MOF/SPGE) on different days using the same fabrication procedure. The R.S.D value for the peak currents obtained for these electrodes in a buffer solution (0.1 M, PBS) containing 50.0 μM of epinine was calculated to be 3.9%, which demonstrates the very good reproducibility of the electrode preparation procedure.

Moreover, the stability of the NiCo-MOF/SPGE was examined by storing the electrode in the lab at room temperature. Then, the electrode was used for the analysis of 50.0 μM of epinine at 1 to 21 days intervals in 0.1 M PBS. The results showed that the electrode signal retained up to 98.7% of its initial value after 7 days and 96.2% of its initial value after 21 days.

### 2.9. Application of the Proposed Sensor for the Determination of Drugs in Real Samples

The developed method can be used successfully for the determination of epinine and venlafaxine in real samples of venlafaxine tablets, urine and water specimens. By using the standard addition method, this study was accomplished and the results of the analysis are shown in Table 1. The appreciable recovery rates (96.8–104.5%) and relative standard deviations (RSDs) (1.6–3.5%) confirmed the capability of NiCo-MOF/SPGE as a voltametric sensor for the analysis of these two drugs in real samples. Also, the reproducibility of the method was characterized using the relative standard deviation (RSD).

## 3. Experimental Section

### 3.1. Instruments and Reagents

An Autolab PGSTAT302N electrochemical device (Eco Chemie, Utrecht, the Netherlands) was employed to conduct electrochemical determinations using the GPES V4.9 software. The electrochemical measurements were obtained with the aid of a three-electrode configuration on graphite SPEs from DropSens (DRP-110, Asturias, Spain). The auxiliary and working electrodes of the SPEs contained graphite ink in contrast, and the pseudo-reference electrode and electric contacts contained silver ink. The solution’s pH was adjusted by a pH-meter (Metrohm 710, Metrohm, Herisau, Switzerland). The deionized water used in each experiment was also taken from a Millipore Direct-Q^®^ 8 UV (ultra-violet) (Millipore, Darmstadt, Germany).

Field-emission scanning electron microscopy (MIRA3TESCAN-XMU) was utilized for morphological studies. Energy-dispersive X-ray spectroscopy (EDS)-FE-SEM (MIRA3TESCAN-XMU) was also applied for elemental analysis. XRD patterns were recorded to obtain the data on structure using a Panalytical X’Pert Pro X-ray diffractometer (The Netherlands) via Cu/Kα radiation (λ = 1.5418 nm). The N_2_ adsorption/desorption isotherms for the bimetallic MOF nanosheets were explored at 77 K using a BELSORP MINI II gas sorption analyzer. The Barrett–Joyner–Halenda (BJH) and Brunauer–Emmett–Teller (BET) approaches were employed to verify the pore diameter and the surface area of the bimetallic MOF nanosheets, sequentially.

Nickel nitrate hexahydrate [Ni(NO_3_)_2_·6H_2_O], cobalt nitrate hexahydrate [Co(NO_3_)_2_·6H_2_O], 2-Aminoterephthalic acid (NH_2_-H_2_BDC) and poly(vinylpyrrolidone) (PVP) were obtained from Sigma-Aldrich (St. Louis, MO, USA). The absolute ethanol and N,N-Dimethylformamide (DMF) came from Merk (Darmstadt, Germany). Venlafaxine, epinine and all other reagents were of an analytical grade (from Sigma-Aldrich). Orthophosphoric acid was used to prepare the phosphate buffer solution (0.1 M, PBS), as a supporting electrolyte.

### 3.2. Fabrication of Bimetallic NiCo-MOF Nanosheets

To this end, Co (NO_3_)_2_·6H_2_O (0.285 mmol, 83 mg) and Ni (NO_3_)_2_·6H_2_O (0.574 mmol, 167 mg) and were dissolved in water (20 mL). Then, ethanol (20 mL) and DMF (20 mL) were poured into PVP (250 mg) plus NH_2_-H_2_BDC (75 mg). The two mixtures were stirred for 30 min to achieve homogeneous and transparent solutions, and were mixed together and stirred in the same way. After 30 min, the obtained solution was placed in a 100-mL Teflon-lined stainless-steel high-pressure autoclave. Subsequently, the autoclave was positioned in an oven for 20 h at 150 °C. Following the reaction, a centrifugation was performed for the appeared product at 10,000 rpm for 15 min using ethanol and DMF to clear the supernatant. The drying of obtained product was performed at 60 °C to collect a purple powder.

### 3.3. Fabrication of Screen-Printed Graphite Electrode Modified with NiCo-MOF

A facile drop-casting protocol was followed to modify the surface of the SPGE with NiCo-MOF nanosheets. Thus, a 1 mg/mL suspension of NiCo-MOF nanosheets underwent a 40-min sonication, and then 5 μL of the suspension was poured onto the SPGE surface, dropwise, followed by drying under room conditions to achieve a NiCo-MOF/SPGE.

The surface areas of the NiCo-MOF/SPGE and the unmodified electrode were obtained by CV using 1.0 mM of K_3_Fe(CN)_6_ at diverse scan rates. Using the Randles−Sevcik formula, in the NiCo-MOF/SPGE, the electrode surface was found to be 0.122 cm^2^, which was approximately 3.9 times greater than that of the unmodified electrode.

### 3.4. Preparation of Real Samples

#### 3.4.1. Pharmaceutical Sample Solution Preparation

Three tablets of the venlafaxine (labeled value of venlafaxine = 75 mg per tablet) purchased from a local pharmacy in Kerman (Iran) were finely powdered in a mortar with a pestle. Then, an accurate weighed amount of the homogenized vanlafaxine powder was transferred into a 100 mL 0.1 mol/L PBS (pH 7.0). The contents of the flask were sonicated for 15 min to affect a complete dissolution. Finally, the solutions were filtered and suitable aliquots of the clear filtrate were collected. Afterward, we poured a determined volume of the transparent filtrate into the electrochemical cell consisting of 25 mL of 0.1 mol/L PBS (pH = 7.0) to record the differential pulse voltammograms.

#### 3.4.2. Preparation of Water Specimens

Tap water was also sampled, filtrated with a membrane filter and poured into 0.1 M PBS (pH = 7.0). At last, the venlafaxine and epinine contents were measured in the water specimens using the as-developed protocol according to the standard addition method.

## 4. Conclusions

A facile protocol was followed to construct bimetallic MOFs with great conductivity for developing electrochemical sensors. The conductive NiCo-MOF nanosheets were applied as an effective electrode material for the modification of the SPGE surface to electrochemically co-detect epinine and venlafaxine in real specimens. The commendable features of the NiCo-MOF nanosheets, based on their surface area, porosity and nanoscale dimension, presented a significant reduction in overvoltage and enhancement in the electrochemical behavior of epinine. A robust current was seen for the as-developed modified electrode in response to the epinine oxidation under the optimized circumstances. According to the findings, the linear dynamic range was as broad as 0.07–335.0 μM, and the limit of detection was as narrow as 0.02 μM in the epinine determination. One of the efficient ways to co-detect the two analytes was found to be the complete resolution of the differential pulse voltammetry peak potentials for venlafaxine and epinine (˃470 mV in separation of peak potentials). The repeatability, reproducibility and stability of the NiCo-metal–organic-framework-nanosheets-modified electrode were investigated, and the relative standard deviations obtained indicated that the NiCo-MOF/SPGE had superior repeatability, reproducibility and stability. Due to its high reliability and rapid determination of epinine and venlafaxine, NiCo-MOF/SPGE is suggested as a potent electrode for sensing these two drugs in real pharmaceutical matrices and tap water samples.

## Figures and Tables

**Table 1:** Determination of epinine and venlafaxine drugs in real samples using NiCo-MOF/SPGE. All concentrations are in µM. (n = 5).

Sample	Spiked		Found		Recovery (%)		R.S.D. (%)	
	Epinine	Venlafaxine	Epinine	Venlafaxine	Epinine	Venlafaxine	Epinine	Venlafaxine
Venlafaxine tablet	0	0		3.2				3.0
	4.0	1.0	4.1	4.1	102.5	97.6	1.9	2.4
	5.0	2.0	4.9	5.3	98.0	101.9	3.2	1.8
	6.0	3.0	6.2	6.0	103.3	96.8	2.6	2.7
	7.0	4.0	6.9	7.4	98.6	102.8	2.3	2.9
Urine	0	0						
	5.0	5.5	4.9	5.7	98.0	103.6	2.8	2.4
	7.0	7.5	7.1	7.3	101.4	97.3	1.9	3.5
	9.0	9.5	8.8	9.6	97.8	101.0	3.2	2.1
	11.0	11.5	11.5	11.4	104.5	99.1	2.7	1.7
Tap water	0	0						
	4.0	4.5	3.9	4.7	97.5	104.4	1.8	3.5
	6.0	6.5	6.1	6.4	101.7	98.5	2.9	2.8
	8.0	8.5	7.9	8.6	98.7	101.2	3.0	1.6
	10.0	10.5	10.4	10.3	104.0	98.1	2.2	2.4

## Data Availability

The data presented in this study are available upon request from the corresponding authors.
